# Primary Mouse Choroidal Endothelial Cell Culture

**DOI:** 10.21769/BioProtoc.5355

**Published:** 2025-06-20

**Authors:** Qiuhua Yang, Yongfeng Cai, Qian Ma, Yuqing Huo

**Affiliations:** 1 Vascular Biology Center, Medical College of Georgia, Augusta University, Augusta, GA, USA; 2 Department of Ophthalmology, Baylor College of Medicine, Houston, TX, USA

**Keywords:** Mouse choroidal endothelial cells, RPE/choroid complex, Matrigel explant culture, CD31^+^ cell enrichment, Endothelial-to-mesenchymal transition (EndMT)

## Abstract

The study of choroidal endothelial cells is essential for understanding the pathological mechanisms underlying choroidal neovascularization and other vision-threatening disorders. Traditional methods for isolating and culturing primary endothelial cells often yield mixed populations or require specialized equipment, limiting their widespread use. Here, we present a straightforward protocol for isolating and culturing primary mouse choroidal endothelial cells. This protocol involves enzymatic digestion of choroidal tissue, magnetic-activated cell sorting (MACS) to enrich CD31^+^ endothelial cells, and optimized culture conditions to promote cell proliferation and maintain endothelial phenotype. The protocol is strategic, reproducible, and requires minimal specialized equipment, making it accessible for researchers across various fields. By providing a robust method for obtaining pure choroidal endothelial cell cultures, this protocol facilitates the study of cell-specific behaviors and responses, advancing research into choroidal vascular diseases.

Key features

• Describes a protocol for isolating mouse choroidal endothelial cells (mCECs) using Matrigel^TM^-based explant culture followed by CD31^+^ cell enrichment.

• Utilizes enzymatic dissociation with dispase and filtration to achieve a single-cell suspension, ensuring high cell yield and purity.

• Confirms endothelial cell identity, enabling reliable downstream applications.

• Supports experimental induction of endothelial-to-mesenchymal transition (EndMT) using mouse transforming growth factor β2 (TGFβ2), making it suitable for studying vascular remodeling processes.

## Graphical overview


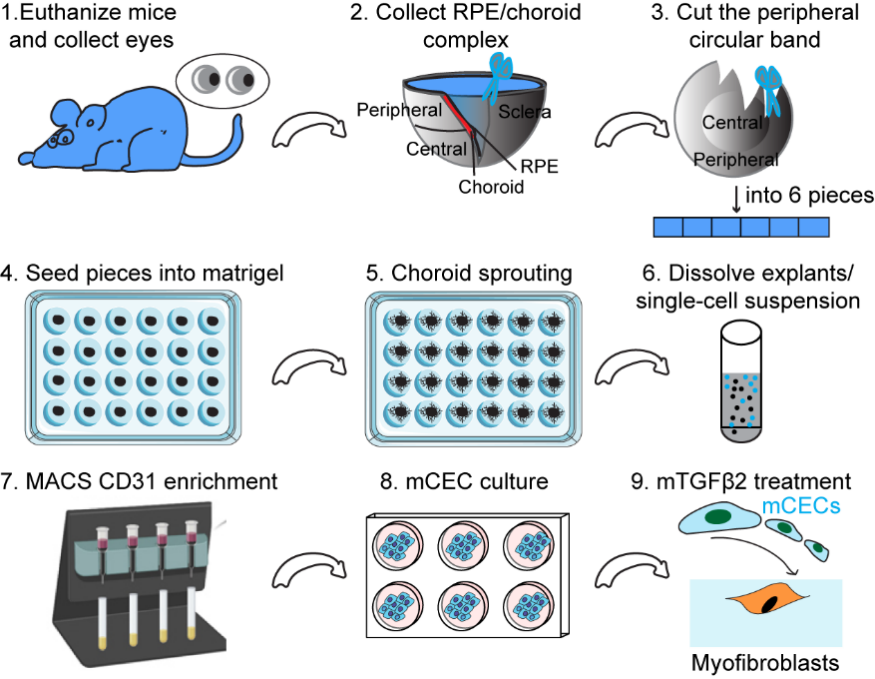


**Schematic illustration of mouse choroidal endothelial cell generation and culture.** This schematic illustration outlines a step-by-step process for isolating and culturing mouse choroidal endothelial cells (mCECs) from the retinal pigment epithelium (RPE)/choroid complex. First, mouse eyes are collected and dissected to isolate the RPE/choroid complex, from which the peripheral choroidal band is cut into small pieces. These tissue pieces are embedded in Matrigel to promote choroidal sprouting, and the resulting explants are enzymatically dissociated into a single-cell suspension. CD31 MicroBeads are used to isolate endothelial cells, which are then cultured in appropriate conditions to expand mCECs. Finally, mCECs are treated with mTGFβ2 to induce their transition into myofibroblasts for further study.

## Background

Choroidal endothelial cells (CECs) are critical for maintaining the vascular integrity of the choroid, which supplies oxygen and nutrients to the outer retina [1,2]. Dysregulation of these cells contributes to the pathogenesis of several ocular diseases, including age-related macular degeneration (AMD) [3], diabetic retinopathy (DR) [4], oxygen-induced retinopathy (OIR) [5], and retinitis pigmentosa (PR) [6], all of which are leading causes of vision loss worldwide. Investigating the molecular mechanisms governing CEC behavior, such as angiogenesis, endothelial-to-mesenchymal transition (EndMT), and interactions with the retinal pigment epithelium, is crucial for developing targeted therapeutic strategies [7]. Thus, establishing a reliable and reproducible method for culturing CECs is critical for studying their role in disease progression.

Researchers began using ex vivo mouse choroidal tissue from isolated choroids in the early 2000s to better understand ocular diseases such as diabetic retinopathy and age-related macular degeneration [8–11]. Currently, multiple assays exist for studying choroidal endothelial cells, including the ex vivo choroid sprouting assay, enzymatic digestion-based isolation, and immunoselection methods. Through the foundational work of previous studies, we have gained valuable insights into the isolation and culture of choroidal endothelial cells. The ex vivo choroid sprouting assay, developed by Shao et al. and Tomita et al., has provided a robust model for studying choroidal microvascular angiogenesis, demonstrating the importance of maintaining a physiologically relevant microenvironment [12,13]. Meanwhile, protocols for endothelial cell isolation from various mouse tissues, including the choroid, outlined by Daneman et al., have highlighted effective strategies for obtaining high-purity endothelial cell populations through enzymatic digestion and immunoselection [14]. In addition, several studies have reported robust methods for isolating endothelial cells and stromal vascular fraction (SVF) cells from other vascular beds, including adipose tissue and the lung, using a combination of mechanical dissociation, enzymatic digestion, and magnetic or flow cytometry–based enrichment [15–17].

Building on these methodologies, we have refined our protocol to enhance the efficiency and specificity of primary mouse choroidal endothelial cell (mCEC) culture. By integrating Matrigel^TM^-based explant culture with CD31^+^ magnetic-activated cell sorting (MACS), we achieve high endothelial cell purity while preserving key functional properties. Additionally, we have optimized culture conditions to balance EC survival and growth while minimizing contamination from other cell types. Although our approach still has limitations, such as the requirement for fresh tissue and extended culture time, it offers a strategic adjustment and reproducible method for studying choroidal endothelial cells at a molecular level. By learning from these established methodologies and making strategic adjustments, our refined protocol provides an improved approach for investigating choroidal endothelial cell biology, facilitating further studies on choroidal angiogenesis and related diseases.

## Materials and reagents

**Biological materials**

1. 6–8-weeks-old C57BL/6J mice (The Jackson Laboratory, Stk#000664)

2. Endothelial cell (EC) lineage tracing mice (*Cdh5* ^Cre^; *Rosa26*-EYFP^f/f^ mice) were generated by crossing *Cdh5* ^Cre^ constitutive mice (The Jackson Laboratory, Stk#006137) with *Rosa26*-EYFP^f/f^ mice (The Jackson Laboratory, Stk#006148)

**Reagents**

**A. Isolation and culture of mouse choroidal endothelial cells**

1. DPBS (Gibco, catalog number: 14190250)

2. Matrigel^TM^ (Corning, catalog number: 354230)

3. Dispase (Corning, catalog number: 354235)

4. EGM^TM^-2 endothelial cell growth medium (Lonza, catalog number: CC-3162)

5. CD31 MicroBeads, mouse (Miltenyi Biotec, catalog number: 130-097-418)

6. Penicillin-streptomycin (Gibco, catalog number: 15-070-063)

7. FcR blocking reagent, mouse (Miltenyi Biotec, catalog number: 130-092-575)

8. Ketamine (Henry Schein Animal Health, catalog number: 071069)

9. Xylazine (Akorn Pharmaceuticals Corporate, catalog number: 033197)

**B. Immunofluorescence staining**

1. 4% paraformaldehyde (PFA) in PBS (Santa Cruz Biotechnology, catalog number: sc-281692)

2. 10% normal goat serum (Thermo Scientific, catalog number: 50062Z)

3. ACTA2 antibody (Santa Cruz Biotechnology, catalog number: sc-32251)

4. Alexa Fluor 594-conjugated goat anti-mouse secondary antibody (Invitrogen^TM^, catalog number: A11032)

5. DAPI and Hoechst nucleic acid stains (Invitrogen^TM^, catalog number: D1306)

6. VECTASHIELD antifade mounting medium (Vector Laboratories, catalog number: H-1000-10)

7. Tween-20 (MP Biomedicals, catalog number: 11TWEEN201-CF)

8. Triton X-100 (Thermo Scientific, catalog number: A16046.AP)

**C. Western blot**

1. RIPA buffer (Sigma-Aldrich, catalog number: R0278)

2. Phosphatase inhibitor (Sigma-Aldrich, catalog number: 4906845001)

3. Protease inhibitor cocktail (Sigma-Aldrich, catalog number: 05892970001)

4. Pierce^TM^ BCA Protein Assay kit (Fisher Scientific, catalog number: 23225)

5. Recombinant mouse TGF-beta 2 protein (R&D Systems, catalog number: 7346-B2-005)

6. ACTA2 antibody (Santa Cruz Biotechnology, catalog number: sc-32251)

7. SM22α antibody (Abcam, catalog number: ab14106)

8. COL1 antibody (Novus Biologicals, catalog number: NB600-408)

9. CD31 antibody (Santa Cruz Biotechnology, catalog number: 376764)

10. β-actin antibody (Santa Cruz Biotechnology, catalog number: sc-47778)

11. Anti-mouse IgG HRP-linked antibody (Cell Signaling Technology, catalog number: 7076S)

12. Anti-rabbit IgG HRP-linked antibody (Cell Signaling Technology, catalog number: 7074S)

13. 10× tris buffered saline (TBS) (Bio-Rad, catalog number: 1706435)

14. Immobilon Forte Western HRP substrate (MilliporeSigma, catalog number: CHM01S430)

15. Non-fat milk (Lab Scientific, catalog number: NC9121673)

**Solutions**

1. PBST buffer (see Recipes)

2. Permeabilization buffer (see Recipes)

3. TBST buffer (see Recipes)

4. 5% non-fat milk (blocking buffer) (see Recipes)

**Recipes**

**1. PBST buffer**

Store at room temperature.

**Table:** 

Reagent	Final concentration	Quantity or Volume
PBS (1×)	1×	500 mL
Tween-20	0.05% (v/v)	250 μL
Total	n/a	500 mL

**2. Permeabilization buffer**

Store at room temperature.

**Table:** 

Reagent	Final concentration	Quantity or Volume
PBS (1×)	1×	500 mL
Triton X-100	0.05% (v/v)	250 μL
Total	n/a	500 mL

**3. TBST buffer**

Store at room temperature.

**Table:** 

Reagent	Final concentration	Quantity or Volume
TBS (10×)	1×	100 mL
Tween-20	0.05% (v/v)	500 μL
H_2_O	n/a	900 mL
Total	n/a	1,000 mL

**4. 5% non-fat milk (blocking buffer)**

Prepare fresh before use.

**Table:** 

Reagent	Final concentration	Quantity or Volume
TBST (Recipe 3)	1×	30 mL
Non-fat milk	5% (w/v)	1.5 g
Total	n/a	30 mL

**Laboratory supplies**

1. 24-well plate (Corning, catalog number: 3524)

2. 40 μm cell strainer (Corning, catalog number: 352340)

3. 35 mm dish (Corning, catalog number: 430165)

4. 8-well cell culture slides (Falcon, catalog number: 354118)

5. 6-well plate (Corning, catalog number: 3516)

6. Microscope slides (Fisher Scientific, catalog number: 12-550-15)

7. Cytiva Amersham^TM^ Hybond^TM^ PVDF membranes (Fisher Scientific, catalog number: 45-004-021)

8. Professional^TM^ Kimtech Science^TM^ Kimwipes^TM^ (Fisher Scientific, catalog number: 06-666)

9. Axygen^TM^ MaxyClear snaplock 1.5 mL microtubes (Fisher Scientific, catalog number: 14-222-155)

10. Falcon^TM^ 15 mL conical centrifuge tubes (Fisher Scientific, catalog number: 14-959-53A)

11. Corning^TM^ centrifuge 50 mL tubes (Fisher Scientific, catalog number: 05-526B)

12. LS columns (Miltenyi Biotec, catalog number: 130-042-401)

13. Iris forceps, curved (World Precision Instruments, catalog number: 15915)

14. Dumont Dumoxel tweezers (World Precision Instruments, catalog number: 14188)

15. Dumont titanium forceps (World Precision Instruments, catalog number: 14187)

16. Noyes micro scissors (World Precision Instruments, catalog number: 500228)

## Equipment

1. Cell incubator (Thermo Scientific, catalog number: 13-998-252L7)

2. Centrifuge (Eppendorf, catalog number: 5430R)

3. ChemiDoc^TM^ imaging system (Bio-Rad, catalog number: 12003153)

4. Inverted Routine Eclipse TS2 microscope for cell morphology (Nikon, catalog number: 3374842)

5. Zeiss 780 inverted confocal for immunofluorescence (Zeiss, catalog number: 420650-9901-000)

6. MACS^®^ MultiStand (Miltenyi Biotec, catalog number: 130-042-303)

7. QuadroMACS^TM^ Separator and Starting kits (Miltenyi Biotec, catalog number: 130-091-051)

8. Microscope for phase contrast photos of individual explants (Leica, model: DMi8)

9. TC20 automated cell counter (Bio-Rad, catalog number: 1450102)

## Software and datasets

1. Image J software (National Institutes of Health; RRID: SCR_003070)

2. Zeiss software (ZEN lite)

3. Image Lab 6.1 (Bio-Rad)

## Procedure

**A. Preparation**

1. Thaw the Matrigel overnight by submerging the vial in ice in a 4 °C refrigerator. Once Matrigel is thawed, swirl the vial to ensure that the material is evenly dispersed.

*Note: Store aliquots in the -20 °C freezer until ready for use. Keep frozen. Aliquoting into one-time-use aliquots will minimize freeze-thaw cycles. Matrigel becomes liquid at 4 °C.*

2. Add 5 mL of penicillin/streptomycin and EGM-2 supplements into 500 mL of EBM-2 to get the complete medium. Aliquot 50 mL of the complete medium to prevent contamination.

3. Aliquot 10 mL of Dispase and store aliquots in the -20 °C freezer until ready for use.

4. Autoclave the scissors and forceps in advance for sterilization. Clean the dissecting microscope and workstation with 75% ethanol. Place a sterile disposable pad to create an aseptic working surface.

*Note: Perform the procedure in a low-traffic room.*

5. Prepare two cell culture dishes (35 mm): one for dissecting eyes and the other for holding eyes.

*Note: Keep them on ice.*

**B. Experimental steps**

1. Euthanize the mice by injection of ketamine (100 mg/kg) and xylazine (10 mg/kg), enucleate the eyes immediately with iris forceps, and keep them in an ice-cold EGM-2 medium before dissection. The choice of medium is identical for both storage and subsequent incubation.

**Critical:** All animal procedures complied with the National Institutes of Health Guide for the Care and Use of Laboratory Animals and were in accordance with the protocol approved by the IACUC (Institutional Animal Care and Use Committee) at Augusta University.

**Figure 1.**
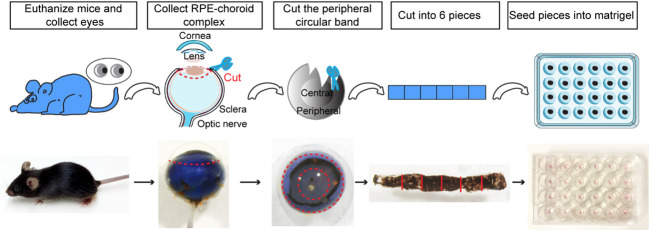
Choroid sprouting assay. First, the eyes were removed, and a circular incision was made behind the limbus. The cornea, iris, lens, and vitreous body were then removed. Next, a 1 mm incision was made from the edge of the eye cup toward the optic nerve. Following this, a circular band was excised approximately 1.0 mm behind the initial incision, and the band, along with the surrounding peripheral tissue, was carefully separated. Finally, the band was cut into approximately 1 mm × 1 mm pieces and embedded in Matrigel^TM^.

2. Using Noyes micro scissors and Dumont Dumoxel tweezers, carefully remove the cornea and the lens from the anterior of the eye. Then, separate the peripheral choroid-scleral complex from the retina with Dumont Dumoxel tweezers. Using the same instruments, cut into approximately 1 mm × 1 mm pieces to obtain choroid/sclera (hereafter referred to as “choroid”) fragments with retinal pigment epithelium (RPE) attached (Figure 1).

*Note: Do not touch the edge.*

**Critical:** Choroid with RPE attached can potentiate endothelial sprouting in EGM-2.

3. Coat the bottom of each well in a 24-well plate with 15 μL of Matrigel^TM^ without touching the edge of the well.

*Note: Matrigel should be kept on ice before use. Ensure that the thickness of the Matrigel^TM^ is approximately 0.4 mm.*

4. Place the choroid fragments in Matrigel^TM^ within the 24-well plate.

*Note: Use one eye for 6 wells, and 2 mice for one 24-well plate.*

5. After seeding the choroid, add an additional 15 μL of Matrigel^TM ^on the top, then incubate the 24-well plate in a 37 °C cell culture incubator without medium for 10 min for the Matrigel^TM^ to solidify (Figure 1).

**Critical:** Ensure choroids expand within the Matrigel^TM^.

**Figure 2.**
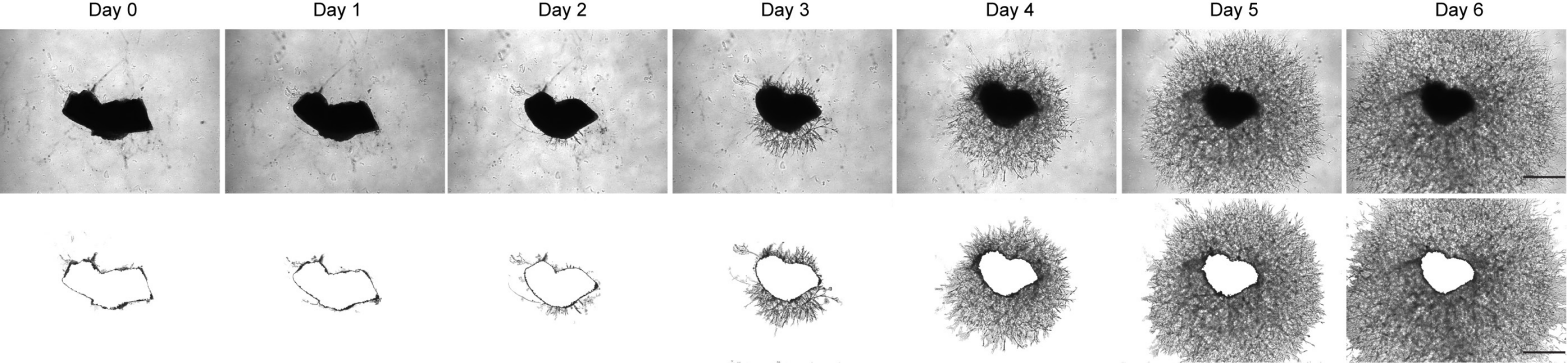
Mouse retinal pigment epithelium (RPE)-choroid sprouting in vitro. Representative images of choroid sprouts at Day 0, 1, 2, 3, 4, 5, and 6. Phase contrast images (top row) show the morphology of mouse choroidal explants and associated sprouting over time. The bottom row displays the corresponding area of vessel outgrowth analyzed using ImageJ, highlighting the region covered by growing vessels. All images were taken from a top-down view. Scale bar = 500 μm.

6. Add 500 μL of EGM-2 medium to each well and incubate at 37 °C with 5% CO_2_ for 48 h before any treatment.

7. Change the medium every 48 h. Take phase contrast photos of individual explants daily using a Leica DMi8 microscope (Figure 2).

8. After 6 days of incubation, discard the medium and wash with plain PBS once, then dissolve the Matrigel^TM^ embedding the choroid explants by adding 500 μL of Dispase per well. Incubate at 37 °C for 2 h.

*Note: Dispase was selected as the dissociation enzyme to detach primary endothelial cells cultured on Matrigel. Its mild proteolytic activity and limited tryptic activity enable effective digestion of basement membrane components such as laminin and collagen IV without reducing cell viability. Compared to trypsin or pepsin, which exhibit strong protease activity and may damage delicate primary cells, Dispase is better suited for preserving the integrity and function of endothelial cells during harvest. Additionally, collagenase alone is less effective in Matrigel digestion due to its specificity for fibrillar collagen rather than basement membrane proteins.*

9. Collect the digested cell suspension from each well into a 50 mL centrifuge tube. Pass the suspension through a 100 μm cell strainer into a 50 mL centrifuge tube to remove undigested tissue fragments and obtain a single cell suspension.

10. Centrifuge at 300× *g* for 5 min.

11. Resuspend the cells with 500 μL of EGM-2 medium. Count the cells using a TC20 automated cell counter.

12. Add 20 μL of FcR blocking reagent per 1 × 10^7^ cells. Vortex briefly, then add 20 μL of CD31 MicroBeads to the mixture and incubate at 4 °C for 15 min.

**Caution:** Due to the extended incubation time, cells may internalize microbeads. To minimize nonspecific uptake, avoid prolonged exposure and perform incubation at 4 °C if possible, or reduce incubation duration as appropriate.

13. Centrifuge cells at 300× *g* for 3 min.

14. Resuspend the cell pellet in 1 mL of EGM-2 medium.

15. Prepare the columns (LS columns) by rinsing with 3 mL of EGM-2 medium.

16. Filter the cell suspension with a 40 μm cell strainer to remove potential cell aggregates and prevent clogging of the column. Apply the cell suspension onto the LS column.

17. Wash the column 3 times with 3 mL of EGM-2 medium.

*Note: Perform washing steps by adding buffer aliquots only when the column reservoir is empty.*

18. Remove the column from the magnetic separator: pipette 5 mL of the EGM-2 medium onto the column (LS). Flush out the magnetically labeled cells by firmly pushing the plunger into the column. Repeat this step for a total volume of 15 mL.

19. Centrifuge at 300× *g* for 5 min.

20. Resuspend the cells in 10 mL of EGM-2 medium to obtain CD31-positive cells.

*Note: Cells can be directly analyzed for purity validation by flow cytometry or taken into culture.*

21. Incubate at 37 °C with 5% CO_2_ and change medium every other day.

## Validation of protocol

**A. Immunofluorescence**

**Figure 3.**
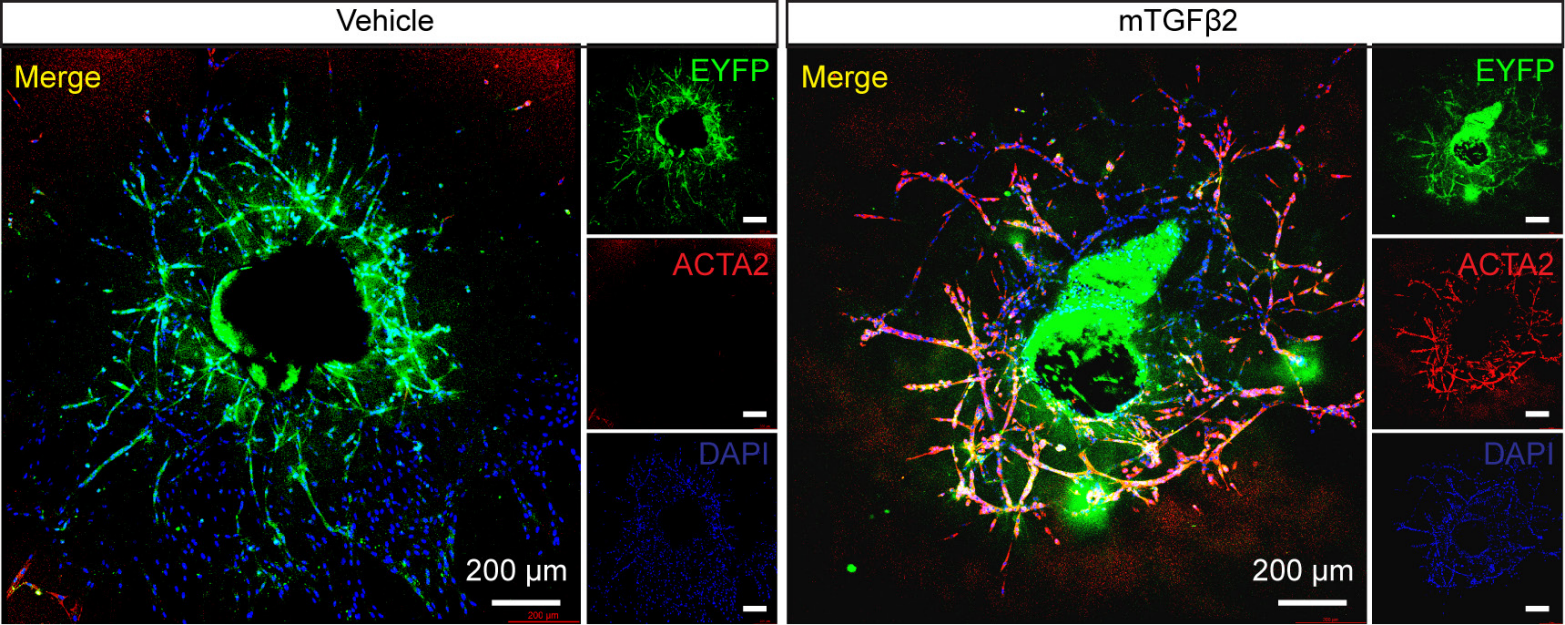
Choroidal sprouting immunostaining. Representative EYFP and ACTA2 immunostaining of choroidal sprouting at Day 6 isolated and cultured from *Cdh5* ^Cre^; *Rosa26*-EYFP^f/f^ mice and treated with or without mTGFβ2 (10 ng/mL) for 3 days. Scale bar, 200 μm.

To characterize the vascular sprouts from the choroid, which exhibit tube-like growth cones of ECs, we cultured sprouts from *Cdh5^Cre^; Rosa26-EYFP^f/f ^* mice. In these mice, ECs are fate-mapped with the *Rosa26-stop-EYFP* (enhanced yellow fluorescent protein) reporter. As shown in Figure 3, ECs were EYFP-positive. After treatment with mTGFβ2 (10 ng/mL) for three days, EYFP-positive ECs expressed the endothelial-to-mesenchymal transition marker ACTA2, suggesting that a subset of ECs had transdifferentiated into mesenchymal cells.

1. Fix the cells on 8-well cell culture slides with 4% (vol/vol) PFA in PBS for 15 min at room temperature (RT). Next, rinse with 1× plain PBS for 5 min at RT.

2. After fixation, permeabilize with permeabilization buffer for 20 min at RT.

3. Block with 10% normal goat serum for 1 h at RT.

4. Incubate with primary antibody against ACTA2 diluted 1/100 (vol/vol) in 10% normal goat serum at 4 °C overnight in a humidified chamber.

5. Remove the primary antibody and wash it three times with PBST for 5 min at RT.

6. Incubate with Alexa Fluor 594-conjugated goat anti-mouse secondary antibody diluted 1/250 (vol/vol) in 10% normal goat serum for 1 h at RT in the dark.

7. Remove the secondary antibody and wash it three times with PBST for 5 min at RT.

8. Incubate the cells for 5 min with DAPI dye (1 μg/mL) to stain the nuclei.

9. Carefully detach the cell chamber and position the glass slide with the cell side facing upward.

10. To mount, pipette a drop of VECTASHIELD antifade mounting medium onto the slide. Carefully place the coverslip over the sample and gently press it down to ensure it lies as flat as possible. Observe the slide using a confocal microscope.

**B. Western blot analysis for endothelial-to-mesenchymal transition markers**

**Figure 4.**
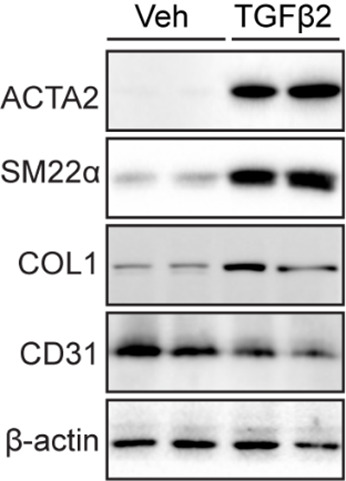
Endothelial-to-mesenchymal transition (EndMT) induction with mouse choroidal endothelial cells (mCECs). Representative western blots of ACTA2, SM22α, COL1, and CD31 protein expression in mCECs isolated and cultured from C57BL/6J mice and treated with or without mTGFβ2 (10 ng/mL) for 5 days.

Western blot analysis was performed to assess protein levels in ECs and evaluate EndMT induction following mTGFβ2 (10 ng/mL) treatment. mCECs exhibited high CD31 expression and low levels of mesenchymal cell markers such as ACTA2, SM22α, and COL1. Upon treatment with mTGFβ2, mCECs showed increased expression of ACTA2, SM22α, and COL1, alongside reduced CD31 expression, compared to vehicle-treated controls (Figure 4).

1. Homogenize cells in RIPA buffer supplemented with 1% phosphatase inhibitor and 1% protease inhibitor cocktail.

2. Measure protein concentration by Pierce^TM^ BCA Protein Assay kit.

3. Load and separate protein by 10% SDS-PAGE, electro-transferred to Cytiva Amersham^TM^ Hybond^TM^ PVDF membranes.

4. Block membranes with 5% non-fat milk buffer for 1 h at RT.

5. Incubate specific antibodies against ACTA2, SM22α, COL1, CD31, and β-actin overnight.

6. Remove the primary antibody and wash it three times with TBST for 10 min at RT.

7. Incubate with HRP-linked IgG secondary antibodies for 1 h at RT.

8. Remove the second antibody and wash it three times with TBST for 10 min at RT.

9. Take images using Immobilon Forte Western HRP substrate with the ChemiDoc MP system.

This protocol or parts of it have been used and validated in the following research article(s):

• Yang et al. [18]. Inactivation of adenosine receptor 2A suppresses endothelial-to-mesenchymal transition and inhibits subretinal fibrosis in mice. *Sci Transl Med*. (Figure S10A, B, and C). PMID: 38446902. DOI: 10.1126/scitranslmed.adk3868.

## General notes and troubleshooting

**General notes**

1. In this protocol, we use mice as an example to isolate choroidal endothelial cells. It also applies to rats.

2. The choroidal explant includes endothelial cells, pericytes, macrophages, and fibroblasts. It is important to use an endothelial cell culture medium.

**Troubleshooting**

Problem 1: Contamination occurs during the culture of mouse choroids.

Possible cause: Contamination introduced during choroid extraction.

Solution: Ensure a sterile working environment, use only sterile instruments and reagents, and add antibiotics (penicillin/streptomycin) to the culture medium.

Problem 2: Choroid tissue floats during initial medium addition or medium change.

Possible cause: The choroid was not properly embedded in Matrigel during placement.

Solution: Use a microscope during the embedding process to confirm that each choroid fragment is fully immersed in Matrigel to prevent floating.

Problem 3: Contamination of cultured mouse CECs with other cell types, such as fibroblasts.

Possible cause: Residual non-CEC cells proliferating due to incomplete purification.

Solution: Perform an additional purification step using CD31 beads to ensure higher purity of CECs.
